# Segmental Renal Infarction Associated with Accessory Renal Arteries After Para-Aortic Lymphadenectomy in Gynecologic Malignancies

**DOI:** 10.3390/medicina61081395

**Published:** 2025-08-01

**Authors:** Ayumi Kozai, Shintaro Yanazume, Fumitaka Ejima, Shuichi Tatarano, Yusuke Kobayashi, Rintaro Kubo, Shinichi Togami, Takashi Yoshiura, Hiroaki Kobayashi

**Affiliations:** 1 Department of Obstetrics and Gynecology, Faculty of Medicine, Kagoshima University, Kagoshima 890-8520, Japan; 2 Department of Perinatology and Gynecology, Faculty of Medicine, Kagawa University, Kagawa 761-0793, Japan; 3 Department of Radiology, Kagoshima University Graduate School of Medical and Dental Sciences, Kagoshima 890-8520, Japan; 4 Department of Urology, Kagoshima University Graduate School of Medical and Dental Sciences, Kagoshima 890-8520, Japan; 5 Course of Advanced Cancer Medicine for Gynecologic Cancer, Graduate School of Medical and Dental Sciences, Kagoshima University, 8-35-1 Sakuragaoka, Kagoshima 890-8520, Japan

**Keywords:** gynecology, kidney, contrast-enhanced computed tomography, para-aortic lymphadenectomy, accessory renal artery, vessel variations, contrast defect

## Abstract

*Background and Objectives*: The causes and clinical outcomes of renal perfusion abnormalities occurring after para-aortic lymphadenectomy (PANDx) for gynecologic malignancies are unknown. We investigated the potential involvement of accessory renal artery (ARA) obstruction in their development by reassessing perioperative contrast-enhanced computed tomography (CECT). *Materials and Methods*: This retrospective study investigated a clinical database to identify urinary contrast defects using CECT in all patients who had undergone PANDx between January 2020 and December 2024. The perfusion defects in the kidney detected by CECT were extracted by a gynecologic oncologist and evaluated by a radiologist and urologist for suspected obstruction of ARAs. *Results*: Postoperative renal contrast defects were observed in 3.8% (6/157) of patients. Renal parenchymal fibrosis, cortical atrophy, and parenchymal thinning were observed as universal findings in all patients showing renal contrast defects. In five of the six cases, ARAs supplying the infarcted renal segments were identified on preoperative CECT, and arterial obstruction was confirmed on postoperative imaging. The remaining case was considered to be latent pyelonephritis. All five patients underwent laparotomy, and preoperative CECT failed to detect ARAs. The median resected para-aortic lymph node was 23 nodes (range: 15–33) in five patients, showing no statistically significant difference compared to patients without perfusion abnormalities (*p* = 0.19). Postoperative serum creatinine levels remained stable. *Conclusions:* ARA obstruction appears to be a risk factor for segmental renal infarction after para-aortic lymphadenectomy in gynecological malignancies; however, the clinical impact on urinary function may be limited. Awareness of this potential complication is essential for gynecologic oncologists performing PANDx.

## 1. Introduction

Para-aortic lymphadenectomy (PANDx) remains an essential surgical procedure for the management of gynecological malignancies. This technique provides critical information for accurate comprehensive staging, guides adjuvant treatment decisions, and may offer therapeutic benefits in selected cases of ovarian and endometrial cancers [1,2]. Despite recent advancements in sentinel lymph node mapping and minimally invasive approaches, comprehensive PANDx continues to be performed in patients with high-risk features or radiologically suspicious para-aortic nodes [3].

The anatomical complexity of the para-aortic region presents a significant challenge during lymphadenectomy. This area contains critical vascular structures including the aorta, inferior vena cava, renal vessels, lumbar vessels, and their branches [4]. Our recent publication reported the incidence of right renal artery injury during robot-assisted PANDx [5]. Smaller branches and anatomical variations may pose considerable surgical risks, and among these, renal artery anomalies deserve special attention because of their frequency and potential clinical impact when injured [4].

Despite the recognized presence of renal vascular anomalies, limited literature exists regarding the clinical impact of PANDx in gynecological malignancies. Few studies have specifically addressed the risk of renal complications associated with inadvertent injury to the aberrant renal vessels during this procedure [6,7]. Moreover, the radiological findings and clinical implications of such injuries remain incompletely characterized in the gynecologic oncology literature.

However, although preoperative prediction of these abnormalities could potentially aid realistic preoperative simulations and reduce surgeons’ burden and perioperative complications, it is difficult to identify variations in the renal vasculature on preoperative contrast-enhanced CT (CEST). We previously constructed preoperative three-dimensional (3D) computed tomography (3DCT) angiography prior to surgery and reported that renal vascular abnormalities were found in 41.2% of patients; in addition, renal vascular and urinary tract abnormalities were clearly demonstrated [8]. Postoperative complications, such as lymphocytic infection, chylous leakage, and intestinal obstruction, were slightly lower in patients who underwent 3DCT. In the above mentioned study, a perfusion defect was observed in the lower pole of the right kidney after surgery in a patient with the most complex urinary tract vascular anomaly, suggesting an obstruction of accessory renal arteries (ARAs) [8].

This current study examined the prevalence, radiological features, and clinical significance of renal perfusion defects following PANDx for gynecological malignancies. Here, we focused specifically on segmental infarction resulting from unrecognized obstruction of ARAs.

## 2. Materials and Methods

### 2.1. Study Design

This retrospective study investigated a clinical database to identify kidney contrast defects using CECT in all patients who had undergone PANDx between January 2020 and December 2024. The study protocol was approved by the Institutional Review Board of Kagoshima University Graduate School of Medical Sciences (approval # 230024,250022). Participants were informed of the study and were offered the opportunity to opt out.

Potential kidney perfusion defects on CECT were extracted by a gynecologic oncologist and evaluated by a radiologist and urologist for suspected obstruction of the ARAs. The following criteria were used to define suspected renal perfusion defects due to obstruction of ARAs, all of which needed to be fulfilled: (1) evidence of ARAs into the infarction site, (2) postoperative obstruction of the responsible ARAs, (3) fibrosis of the renal parenchyma, and (4) atrophy and thinning of the renal parenchyma. Regarding terminology, perfusion defects of the kidney by CECT were defined as a significant reduction in contrast enhancement compared with preoperative CECT, and renal infarction was defined as the presence of fibrosis, atrophy, and thinning.

### 2.2. CT Scan Details

Two CT protocols were employed, one with an arterial phase (Protocol A) and one without (Protocol B). A nonionic contrast agent (Ioversol 320 mg I/mL, Optiray^®^, Guerbet LLC, Princeton, NJ, USA) was used in both protocols, with an injection volume of 1.8 mL/kg body weight up to a maximum of 100 mL. The contrast material was followed by a 20 mL saline flush administered at the same injection rate. In Protocol A, the injection rate was set at an injection volume/30 s, and arterial phase scans were initiated 18 s after the attenuation in the abdominal aorta increased to 150 HU, as measured by a dedicated monitoring system. In Protocol B, the injection rate was fixed at 1.5 mL/s, and no arterial phase was included. For both protocols, parenchymal and excretory phase scans were acquired at 80 s and 300 s after contrast injection, respectively. Three-dimensional reconstructions of the renal arteries, renal veins, and urinary tract were created using a workstation (Ziostation2; Ziosoft Inc., Tokyo, Japan) and fused to enhance visualization.

Commonly performed surgical techniques in our standard surgical techniques for endometrial and ovarian cancers typically include peritoneal cytology, hysterectomy, bilateral salpingo-oophorectomy, PANDx, pelvic lymphadenectomy, and, when necessary, omentectomy or appendectomy. In endometrial cancer, radical or semi-radical hysterectomy was performed when cervical invasion was anticipated. For cervical cancer, radical hysterectomy was performed, with PANDx performed only when metastasis to the higher para-aortic lymph nodes was expected. PANDx was defined as the resection of the lower edge of the left renal vein. Clinical data were collected by reviewing the inpatient medical records. Robotic surgeries were performed using the Da Vinci Xi Surgical System (Intuitive Surgical Inc., Sunnyvale, CA, USA). Surgeries were performed by four gynecologic oncologists certified by the Japan Society of Gynecologic Oncology, employing surgical techniques that have been previously documented in the literature [5], regardless of whether standard laparotomy or minimally invasive surgery was performed. For urinary vessel variation, a supplemental renal artery that originated from any region around the aorta was defined as an accessory renal artery (ARA).

### 2.3. Statistical Analysis

Student’s *t*-test and the Mann–Whitney U test were used to evaluate statistical significance of the differences between groups. Statistical significance was set at *p* < 0.05. All statistical analyses were performed on a personal computer using a statistical software package (SPSS for Windows, v.29; SPSS Inc., Chicago, IL, USA).

## 3. Results

Table 1 shows the demographic and clinical characteristics of the patients who underwent PANDx as part of the initial treatment. Endometrial cancer was the most common, and metastasis to the para-aortic lymph nodes was found in 12.7% of patients. Among patients with endometrial cancer, 45.5% (45/99) had a high-risk histological type. CECT demonstrated a sensitivity of 41.7% and specificity of 96.7% for detecting para-aortic lymph node metastasis and a sensitivity of 28.6% and specificity of 93.8% for pelvic lymph node evaluation.

**Figure 1:**
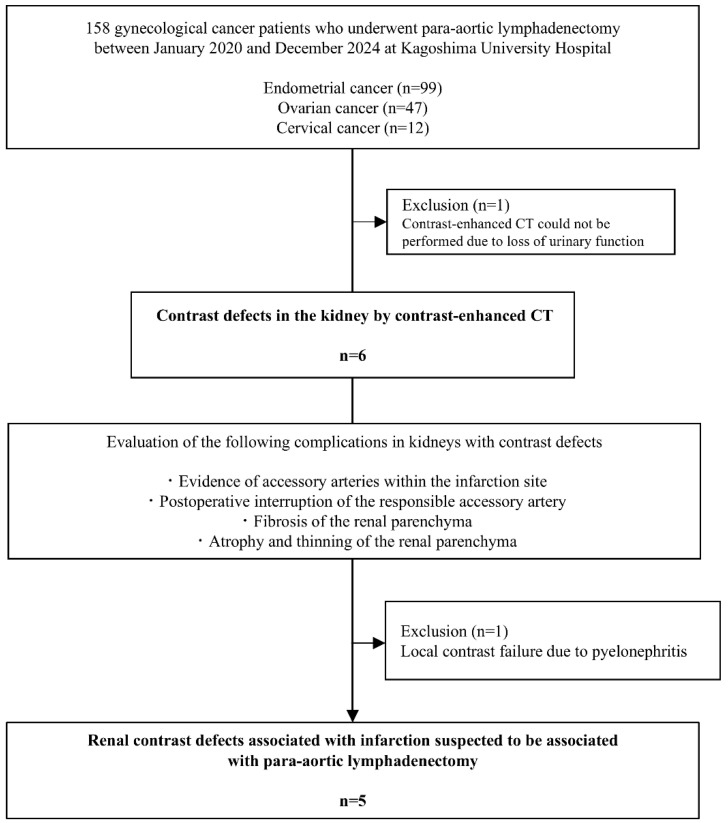
Flowchart summarizing the current study. Abbreviation: CT, computed tomography.

Figure 1 shows the study flowchart and the patient selection process. One patient with renal impairment who did not undergo CECT was excluded, leaving 157 patients in the final analysis. Postoperative renal perfusion defects were observed in 3.8% (6/157) of patients.

**Figure 2:**
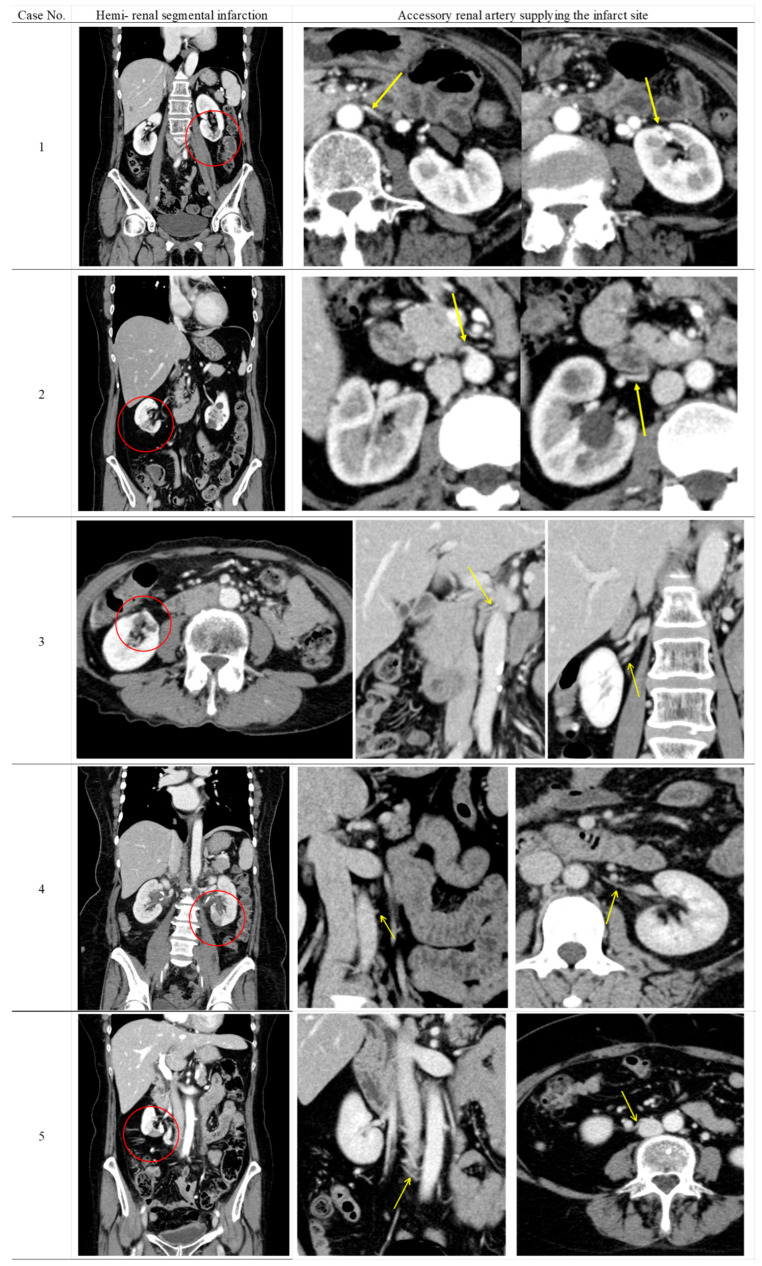
Contrast-enhanced computed tomography findings in five patients with renal perfusion defect potentially involving accessory renal artery obstruction.

Table 2 shows the radiological findings and perioperative serum creatinine levels of patients who developed renal perfusion defects following surgery. Furthermore, the CECT findings of five patients with suspected renal perfusion defects due to ARA obstruction are shown in Figure 2. None of the six patients had clinical or laboratory findings of urinary tract infection at the time of CEST when perfusion defects were detected. Renal parenchymal fibrosis, cortical atrophy, and parenchymal thinning were observed as universal findings in all patients showing renal contrast defects.

In five of the six cases, ARAs supplying the infarcted renal segments were identified on preoperative CECT, and arterial obstruction was confirmed on postoperative imaging. Three of the five patients had an infarction in the lower pole of the kidney. These findings indicated that surgical intervention was the cause of segmental renal infarction. The remaining case was considered latent pyelonephritis due to the absence of identifiable collateral circulation and minimal parenchymal changes.

Preoperative CECT failed to detect any renal vascular anomalies. Table 3 summarizes the clinical profiles of patients who developed CECT-evident perfusion defects. While perioperative serum creatinine levels remained stable between baseline and postoperative day seven (*p* = 0.572), long-term follow-up revealed significantly elevated Cr values compared with preoperative levels at 1 year (*p* = 0.015) and 2 years (*p* = 0.0048), demonstrating progressive renal function deterioration. Endometrial cancer was predominant, with three patients presenting with radiographically suspicious para-aortic lymphadenopathy. All patients underwent laparotomy. The median number of resected para-aortic lymph nodes was 23 (range: 15–33), showing no statistically significant difference compared with patients without perfusion abnormalities (*p* = 0.19).

## 4. Discussion

PANDx is an essential surgical technique in gynecologic oncology, particularly for accurate staging and treatment planning for gynecologic malignancies. Although its therapeutic and prognostic value is well established, ARA management associated with this procedure is not well understood. In our institution, partial renal perfusion defects were observed on postoperative CECT in 3.8% (6/157) of patients who underwent PANDx, and ARA obstruction was observed in five of six patients. None of the five exceptions had ARAs identified prior to surgery. A previous similar report has identified the presence of partial hemi-renal infarction after PANDx, attributing the cause to supernumerary coagulation around the renal artery [9]. We clarified that this phenomenon was potentially caused by intraoperative obstruction of ARAs.

In addition to ARA and main renal artery injury, the most common cause for failure of renal parenchyma perfusion at the normal time on CECT is urinary obstruction, which may be caused by compression of the ureter due to lymphatic fluid retention or scarring after PANDx. The next most common cause is renal vein thrombosis, which may develop due to surgical procedures or postoperative venous stasis. Other causes include external compression and postoperative lymphocele or hematoma formation, which compress the kidney [10]. However, none of these conditions were observed in any of the patients with contrast defects in the current study.

The incidence of renal artery variations has been reported to range between 10 and 50% in the general population, with approximately 30% of individuals having at least one additional renal artery [4,6,8,11]. In our study, the identification of ARA in all five cases of postoperative renal infarction highlighted the clinical significance of these anatomical variations during PANDx.

ARAs are particularly important in this context, because they sometimes function as end arteries without collateral circulation [6]. Our findings demonstrated that inadvertent injury to these vessels during lymphadenectomy led to segmental renal infarction, which was evident on postoperative imaging as areas of decreased enhancement with subsequent thinning and atrophy of the affected renal parenchyma. This observation is consistent with previous reports suggesting that ligation or transection of the polar renal arteries can cause infarction in approximately 30% of the renal parenchyma [12].

Several case reports have described patients in whom the accessory polar renal artery or abnormal renal artery branches were not identified intraoperatively and were injured during PANDx [13].

The clinical implications of such injuries extend beyond the immediate post-operative period. While all patients in our series remained asymptomatic during the immediate postoperative period, our long-term follow-up analysis revealed statistically significant deterioration in renal function at 1 year and 2 years postoperatively compared to baseline values. This finding demonstrates that the observed perfusion defects have measurable clinical impact beyond radiological abnormalities. The literature suggests potential long-term consequences including renovascular hypertension, progressive renal dysfunction, and irreversible damage to renal parenchyma [14], which our findings support through objective measurement of declining renal function over time. Eitan et al. previously reported a case of lower pole renal infarction following ARA injury during PANDx for endometrial cancer [7], emphasizing the real-world risk of this complication.

ARA preservation is crucial for minimizing renal parenchymal damage. In a study [15] comparing the results of CT image interpretation by 11 radiologists (with varying levels of experience) with surgical findings in 94 living kidney donors, five of seven ARAs initially missed on CT measured 2.0–2.5 mm, and two measured 1.5 mm in size. All seven ARAs missed during the initial interpretation were correctly identified during the retrospective review of transverse CT scans. The fact that all vascular abnormalities were potentially detectable in retrospective review by a professionally experienced reader also suggests the value of professional experience. Even more remarkable was the fact that only three of the seven overlooked ARAs were confirmed in the 3D images. While 3DCT reconstruction has been advocated for identifying ARAs prior to PANDx, small accessory vessels that can result in segmental renal infarction may not be reliably detected even with advanced imaging techniques. This emphasizes the importance of prevention through anatomical knowledge and careful surgical techniques. Therefore, during procedures, meticulous dissection and careful handling of tissues in the para-aortic region, particularly around the renal vessels, is paramount. A critical limitation in our study was that preoperative CECT failed to identify ARAs in all cases where postoperative renal infarction occurred. This finding highlights an important gap in routine radiological assessment and clinical communication. The detection of ARAs on standard CECT requires specialized expertise in renal vascular anatomy and dedicated attention to these structures, which may not be routinely provided unless specifically requested by the clinician. Without explicit clinical indication or specific request for ARA evaluation, radiologists may not systematically assess for these anatomical variations in routine abdominal imaging reports.

Therefore, we recommend that gynecologic oncologists specifically request evaluation of renal vascular anatomy, including the presence and course of ARAs, when ordering preoperative CECT for patients scheduled for PANDx. This targeted approach would ensure that radiologists focus on identifying these clinically relevant anatomical variations and provide appropriate comments regarding their presence, size, and potential surgical implications. Enhanced communication between surgical and radiology teams regarding the clinical significance of ARA identification could significantly improve preoperative planning and potentially reduce the risk of inadvertent vascular injury during PANDx.

To our knowledge, this represents the first systematic institutional analysis specifically examining the incidence and characteristics of renal perfusion defects following PANDx for gynecological malignancies. Our comprehensive literature search revealed a notable absence of comparable studies from other institutions or countries that have systematically investigated this complication using similar methodology and diagnostic criteria. The existing literature primarily consists of isolated case reports describing individual instances of renal artery injury during PANDx [7,9,13], rather than systematic institutional analyses of the broader spectrum of renal perfusion abnormalities.

This lack of comparable data presents both opportunities and limitations for our study. While it highlights the novelty of our systematic approach to identifying and characterizing this complication, it also limits our ability to compare our observed incidence of 3.8% with other institutional experiences or validate our findings across different surgical populations and techniques. The absence of similar studies may reflect under-recognition of this complication at other institutions.

Our study has some limitations. First, it was retrospective and had a relatively small sample size. Second, variations in surgical techniques among different surgeons might have influenced the risk of vascular injury. However, vascular injuries are likely to occur with a surgeon’s first 10 cases [16], so technical inexperience was unlikely to be a factor in our research. Third, even if fibrosis occurs due to renal infarction, it may not necessarily indicate poor contrast enhancement; therefore, perfusion defects on CECT may not always accurately reflect renal damage [17]. The retrospective design introduces several potential biases that may affect the interpretation of our findings. Information bias may have influenced our results due to incomplete documentation of surgical details, anatomical variations encountered during surgery, or intraoperative complications. Temporal bias may exist as cases were collected over a 5-year period during which surgical techniques, imaging protocols, and perioperative management may have evolved, potentially affecting the consistency of outcomes and risk assessment. Additionally, measurement bias may be present as the evaluation of renal perfusion defects may be subject to inter-observer variability in the interpretation of CECT findings and the determination of their relationship to ARA obstruction. This study had a relatively small sample size, which limits the statistical power to identify risk factors and may not capture the full spectrum of this complication. Finally, our study did not include systematic assessment of patient-reported quality of life measures or detailed evaluation of how renal perfusion defects might have influenced subsequent treatment decisions, such as modifications to nephrotoxic chemotherapy regimens or considerations for future surgical interventions. The clinical impact of these findings on patient-centered outcomes remains to be fully characterized.

## 5. Conclusions

In conclusion, our study highlighted the significance of renal artery anomalies as risk factors for segmental renal infarction following PANDx in gynecologic malignancies. We recommend thorough preoperative vascular assessment by CECT with or without 3D-CT when feasible, with specific clinical requests for ARA evaluation to ensure focused radiological attention to these anatomical variations, and meticulous attention should be paid to vascular anatomy during dissection. Improved communication between gynecologic oncologists and radiologists regarding the clinical significance of ARA identification is essential for optimal preoperative planning. Our long-term follow-up analysis demonstrated statistically significant deterioration in renal function at 1 and 2 years postoperatively, indicating that these perfusion defects have measurable clinical impact beyond radiological findings. While patients remained asymptomatic, the progressive decline in renal function may have implications for future treatment decisions and long-term patient care. Awareness of this potential complication is essential for gynecologic oncologists performing PANDx. Prospective studies with larger sample sizes, standardized imaging protocols, systematic documentation of intraoperative findings, and standardized radiological assessment criteria would provide more definitive evidence regarding the incidence, risk factors, and long-term consequences of this complication.

## Figures and Tables

**Table 1:** Clinical characteristics of the study including 157 patients in para-aortic lymphadenectomy.

			*n* = 157	
* Age, years (range)			58	(29–78)
* Height, cm (range)			155	(140–170)
* Body weight, kg (range)			55	(37–100)
* BMI (range)			22.7	(15.9–37.1)
Disease				
	^†^ Endometrial cancer		99	(63.1%)
	^‡^ Ovarian cancer		46	(29.3%)
	^§^ Cervical cancer		12	(7.6%)
Stage				
	I–II		107	(68.2%)
	III–IV		50	(31.8%)
Surgery				
	Laparotomy		141	(89.8%)
	Laparoscopy		10	(6.4%)
	Robot		6	(3.8%)
Treatment				
	Simple hysterectomy ± BSO + RPLD		103	(65.6%)
	Semi-Radical hysterectomy ± BSO + RPLD		33	(21.0%)
	Radical hysterectomy ± BSO + RPLD		16	(10.2%)
	Others including RPLD		5	(3.2%)
Number of resected lymph nodes				
	Para-aorta (range)		17	(2–45)
	Pelvis (range)		30	(8–60)
Omentecotomy				
	Yes		103	(65.6%)
	No		54	(34.4%)
Lymph node metastases by histology				
	Pelvis	Yes	25	(15.9%)
		No	132	(84.1%)
	Para-aorta	Yes	20	(12.7%)
		No	137	(87.3%)
Lymph node metastases suspected by computed tomography				
	Pelvis	Yes	21	(13.4%)
		No	136	(86.6%)
	Para-aorta	Yes	22	(14.0%)
		No	135	(86.0%)
Adjuvant therapy				
	None		18	(11.5%)
	Chemotherapy		128	(81.5%)
	Chemoradiotherapy		7	(4.5%)
	Others		4	(2.5%)
Administering bevacizumab after the surgery				
	Yes		10	(6.4%)
	No		147	(93.6%)

* All data are indicated in median values. Diseases were classified according to the FIGO staging system of † 2018, ‡ 2020, and § 2018. Abbreviations: BMI, body mass index; BSO, bilateral salpingo-oophorectomy; RPLD, retroperitoneal lymphadenectomy.

**Table 2:** List of patients with renal perfusion defects detected by CT.

Case No.	Contrast-Enhanced CT Findings					Serum Creatinine Levels			
	Renal Fibrosis	Renal Atrophy and Thinning	Branch Level of Accessory Renal Artery Flowing into the Renal Perfusion Defect	Distribution	Postoperative Obstruction of the Responsible Accessory Renal Artery	Pre-Operative	Post-Operative Day 7	Post-Opeative A Year	Post-Operative Two Years
1	±	+	L2/3	Lower anterior region of the left kidney	+	0.49	0.59	0.66	0.63
2	+	+	L2/3	Anterior ventral region of the right kidney	+	0.73	0.82	0.93	0.94
3	+	+	L2	Anterior ventral region of the right kidney	+	0.61	0.56	0.64	0.70
4	+	+	L2/3	Lower anterior inner region of the left kidney	+	0.54	0.55	0.69	0.71
5	+	+	L4	Lower pole of the right kidney	+	0.7	0.65	0.78	0.78
6	+	+	Nil	Nil	Nil	N/A	N/A	N/A	N/A

Statistical analysis: Preoperative vs. postoperative day 7: *p* = 0.572; Preoperative vs. 1 year: *p* = 0.0154; Preoperative vs. 2 years: *p* = 0.0048. Abbreviation: CT, Computed tomography; L, lumbar spine; N/A, not applicable.

**Table 3:** Clinical backgrounds of the 5 patients with contrast defect of CT.

Patient No.	Age, Years	BMI	Disease	Stage	Para-Aortic Lymph Node Metastases Suspected by CT	Surgery	Treatment	Number of Resected Para-Aortic LN	Para-Aortic LN Metastases	Adjuvant Therapy	Rec	Death
1	56	20.4	^†^ Endometrial ca	IA	Yes	Laparotomy	Simple hysterectomy ± BSO + RPLD	23	Negative	No	No	No
2	53	17.8	^‡^ Cervical ca	IIIC2	Yes	Laparotomy	Radical hysterectomy ± BSO + RPLD	16	Positive	Yes	No	No
3	62	17.5	^†^ Endometrial ca	IB	No	Laparotomy	Semi-Radical hysterectomy ± BSO + RPLD	25	Negative	Yes	Yes	Yes
4	51	35.7	^†^ Endometrial ca	IIB	Yes	Laparotomy	Simple hysterectomy ± BSO + RPLD	33	Negative	Yes	No	No
5	69	24.7	^†^ Endometrial ca	IA	No	Laparotomy	Simple hysterectomy ± BSO + RPLD	15	Negative	Yes	No	No

Diseases were classified according to the FIGO staging system of † 2018 and ‡ 2018. Abbreviations: CT, computed tomography; BMI, body mass index; BSO, bilateral salpingo-oophorectomy; RPLD, retroperitoneal lymphadenectomy; LN, lymph node; Rec, recurrence.

## Data Availability

The data for this study are shown in tables and figures; no other datasets were generated or analyzed during the current study.
